# Enteric Species F Human Adenoviruses use Laminin-Binding Integrins as Co-Receptors for Infection of Ht-29 Cells

**DOI:** 10.1038/s41598-018-28255-7

**Published:** 2018-07-03

**Authors:** Anandi Rajan, B. David Persson, Lars Frängsmyr, Annelie Olofsson, Linda Sandblad, Jyrki Heino, Yoshikazu Takada, A. Paul Mould, Lynn M. Schnapp, Jason Gall, Niklas Arnberg

**Affiliations:** 1 Department of Clinical Microbiology/Virology, and, the Laboratory for Molecular Infection Medicine Sweden, Umeå University, Umeå, Sweden; 2 Department of Molecular Biology, Umeå University, Umeå, Sweden; 3 Department of Biochemistry, University of Turku, Turku, Finland; 4 Department of Dermatology, Biochemistry and Molecular Medicine, UC Davis School of Medicine, California, USA; 5 Biomolecular Analysis Core Facility, Faculty of Biology, Medicine and Health, University of Manchester, Manchester, United Kingdom; 6 Division of Pulmonary, Critical Care, Allergy and Sleep Medicine, Medical University of South Carolina, Charleston, USA; 7 Vaccine Research Center (VRC), NIAID, NIH, Bethesda, USA

## Abstract

The enteric species F human adenovirus types 40 and 41 (HAdV-40 and -41) are the third most common cause of infantile gastroenteritis in the world. Knowledge about HAdV-40 and -41 cellular infection is assumed to be fundamentally different from that of other HAdVs since HAdV-40 and -41 penton bases lack the αV-integrin-interacting RGD motif. This motif is used by other HAdVs mainly for internalization and endosomal escape. We hypothesised that the penton bases of HAdV-40 and -41 interact with integrins independently of the RGD motif. HAdV-41 transduction of a library of rodent cells expressing specific human integrin subunits pointed to the use of laminin-binding α2-, α3- and α6-containing integrins as well as other integrins as candidate co-receptors. Specific laminins prevented internalisation and infection, and recombinant, soluble HAdV-41 penton base proteins prevented infection of human intestinal HT-29 cells. Surface plasmon resonance analysis demonstrated that HAdV-40 and -41 penton base proteins bind to α6-containing integrins with an affinity similar to that of previously characterised penton base:integrin interactions. With these results, we propose that laminin-binding integrins are co-receptors for HAdV-40 and -41.

## Introduction

HAdVs are classified into species A to G and the yet increasing number of HAdV types^1^ cause disease mainly in airways (species A, B, C and E), eyes (species B, C, D and E), urinary tract (species B) and gastrointestinal tract (species A, C, D, F, G)^2^. Whereas most other HAdVs exhibit a broad tropism, enteric species F HAdV-40 and -41 exclusively cause gastroenteritis and are a major cause of infantile gastroenteritis worldwide after rotavirus and norovirus^3,4^, with a seroprevalence greater than 40%^4^.

Most HAdVs infect host cells through a two-step process. First, the knob domain of the trimeric fibre capsid protein interacts with primary cellular receptors, such as the coxsackie and adenovirus receptor (CAR)^5^, sialic acid-containing glycans^6^, CD46^7–9^, and desmoglein 2 (DSG-2)^10^. All HAdVs are equipped with one single fibre protein except for species F HAdV-40 and -41 and species G HAdV-52, which are equipped with one long, CAR-binding fibre and one short fibre^5,11–13^. The second step involves the interaction of the penton base (PB) capsid protein to secondary or co-receptors on the cell, which leads to internalisation and endosomal escape^14^. Species A to E HAdVs use the RGD-binding group of integrins as co-receptors^14–19^. Integrins are transmembrane, heterodimeric glycoproteins involved in signalling, cell adhesion and cell migration^20^. The dimers are built by the non-covalent association of one α and one β polypeptide, and are classified into four groups based on their ligand interactions^21^. LDV (leucine-aspartate-valine)-binding integrins (α4β1, α4β7, α9β1, αEβ7 and the β2-containing subgroup including αXβ2, αMβ2, αLβ2, αDβ2) interact with ligands on epithelial cells or on leukocytes, such as VCAM-1 (vascular cell adhesion molecule 1) and MAdCAM-1 (mucosal vascular addressin cell adhesion molecule 1) and are involved in cell-cell interactions and migration (leukocyte homing). The αV integrins (αVβ1, αVβ3, αVβ5, αVβ6, αVβ8), α5β1, α8β1 and αIIbβ3 are known as RGD (asparagine-glycine-aspartate)-binding integrins. These integrins are involved in cell-matrix adhesion and interact with extracellular matrix (ECM)-containing proteins such as vitronectin and fibronectin. The two remaining groups, which partly overlap, include collagen-binding integrins (α1β1, α2β1, α10β1 and α11β1) and laminin-binding integrins (α1β1, α2β1, α3β1, α6β1, α6β4 and α7β1). These groups are also involved in cell matrix adhesion.

Interactions of HAdV species A to E with integrins are mediated by the exposed RGD-containing loops found in each monomer of the pentameric PB. Carrying the fibres, the PBs are located at each of the 12 vertices of an icosahedral HAdV particle^22^. The PBs of species F HAdVs are unique in that they lack the otherwise conserved RGD motif (present in all other HAdVs), which mediates the interaction with integrins, and are instead replaced with a RGAD motif in HAdV-40 and an IGDD motif in HAdV-41^23^. Combined with the exclusive gastrointestinal tropism and the nearly unique presence of two fibres in these HAdVs, it has been suggested that the short fibres have replaced the entry function of the PBs^5,24^. In this study, we challenged this suggestion and hypothesised that the PB proteins of these HAdVs contribute to cellular entry through interactions with laminin-binding integrins.

## Results

### Multiple α integrin subunits promote HAdV-41 transduction of CHO cells

**Figure 1:**
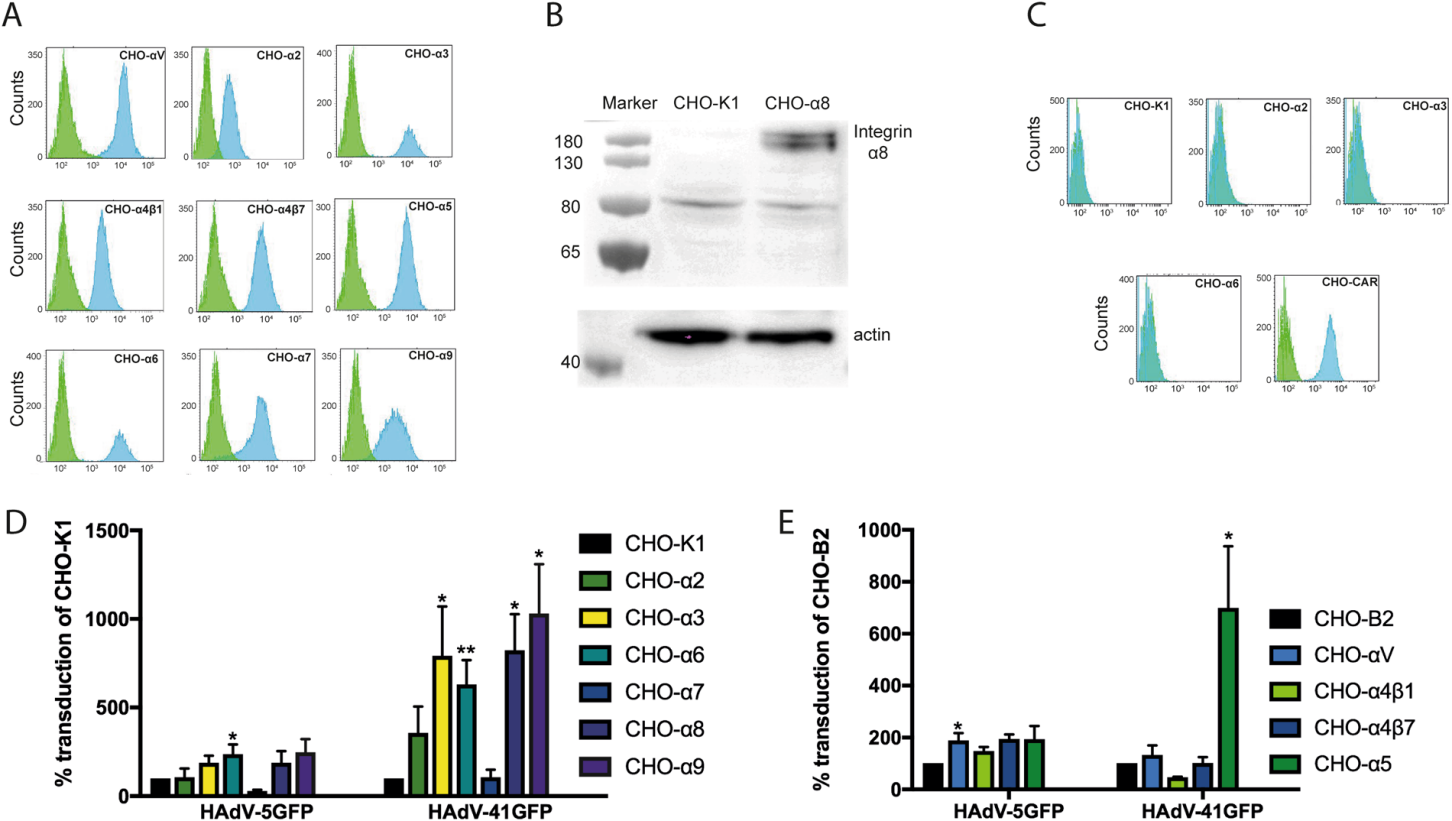
HAdV vector transduction of CHO-cells expressing human alpha integrin subunits. (**A**) Blue histograms show expression of corresponding alpha subunit of human integrin cDNA-transfected cells as compared to expression on control cells (green histograms). Fluorescence intensity is displayed on x-axes and counts are displayed on y-axes. (**B**) Western blot analysis of α8 integrin in transfected CHO cells and control CHO-K1 cells. Cropped blots are shown for each protein evaluation (Full-length blots of each tested protein are reported in Figure S1). (**C**) Flow cytometry with anti-CAR mAb on CHO-integrin and CHO-CAR cells. CHO-integrin cells lack expression of human CAR as shown by blue histograms as compared to only 2° antibody (green histograms). Transduction of CHO integrin cells lacking or expressing human integrins by GFP encoding vectors - HAdV-5 or HAdV-41 with (**D**) CHO-K1 as a control cell line and (**E**) CHO-B2 as a control cell line. The data are represented as % transduction of the control cells. Experiments were performed three times with duplicate samples in each experiment. Error bars in (**C**) and (**D**) represent mean ± SEM. *P of <0.05 and **P of <0.01 versus control.

To study the relevance of non-RGD binding integrins during entry and infection by enteric HAdVs, we used a library of CHO (Chinese hamster ovary) cell lines^25^ that overexpress human integrin alpha subunits. Flow cytometry (Fig. 1A) and western blot (Fig. 1B) analyses confirmed the expression of each integrin on the respective CHO cell type. Western blot was performed to analyse expression levels of α8 since we could not identify a flow cytometry-compatible antibody for this subunit. These CHO cells do not express CAR as shown by flow cytometry with an anti-human CAR antibody **(**Fig. 1B**)**. To examine which integrins HAdV-41 uses to enter these cells, we used a GFP-encoding HAdV-41 vector (HAdV-41GFP). The vector transduced CHO cells that expressed the laminin- and/or collagen-binding α2, α3, and α6 subunits, the RGD-binding α5 and α8 subunits or the LDV-binding α9 subunit more efficiently than the respective control CHO cells (Fig. 1C,D). There was no enhanced transduction in CHO cells expressing αV, α4, and α7 subunits. In agreement with previous data on HAdV-5^14^, we found that GFP-encoding HAdV-5 (HAdV-5GFP), which was used here as a reference vector, transduced CHO-αV cells more efficiently than control cells. The increased transduction of CHO cells expressing human laminin-, collagen-, RGD-, and LDV-binding integrins by HAdV-41GFP indicated that these integrins could potentially play a role in the early steps of HAdV-41 infection. Since the enteric HAdV PB do not contain RGD motifs, but instead contain potential laminin-binding integrin motifs, also in the otherwise RGD-containing loops, we focused on investigating the role of laminin-binding integrins.

### Soluble laminins prevent HAdV-41 infection and internalisation in HT-29 cells

**Figure 2:**
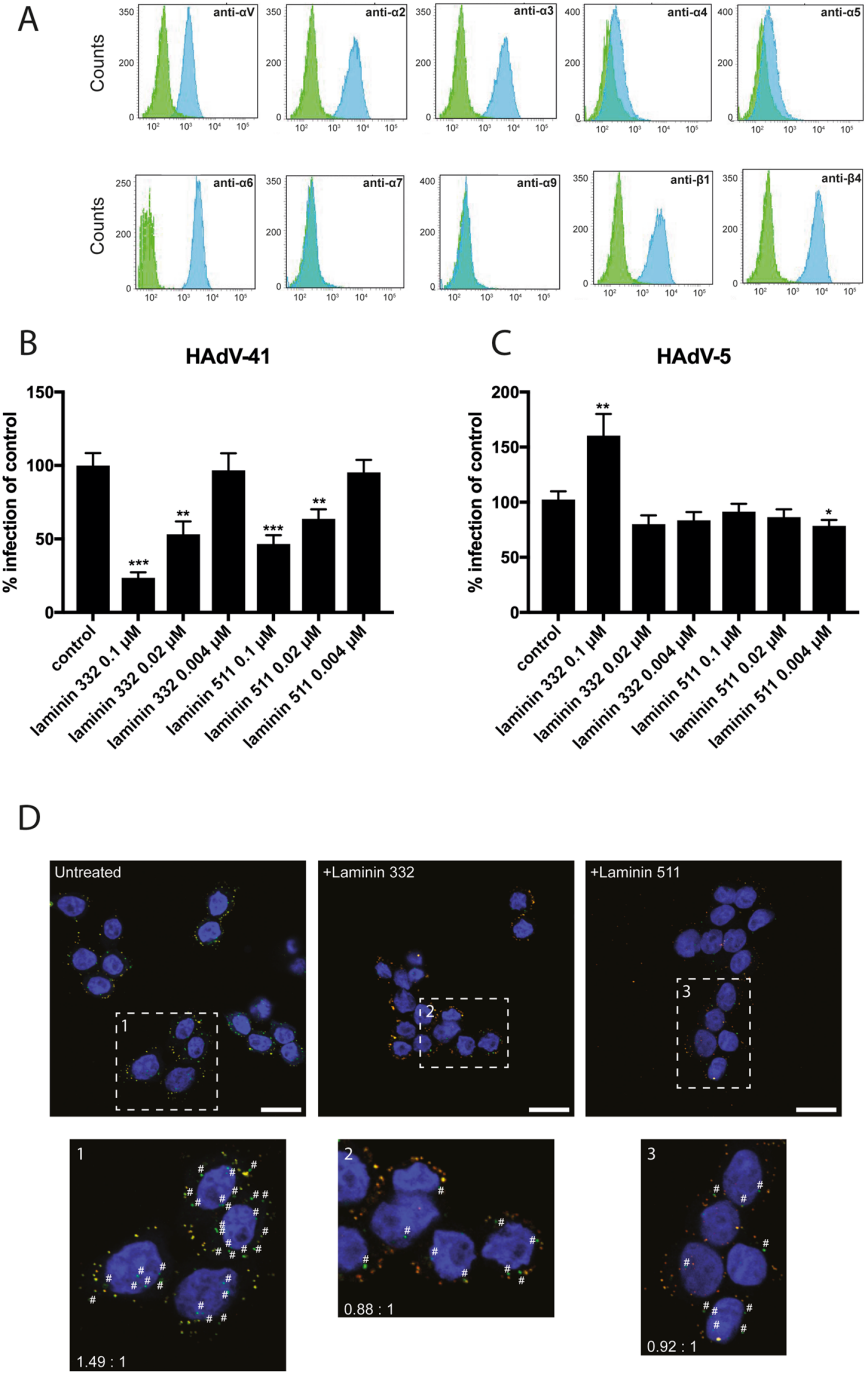
HAdV infection of HT-29 cells in presence of soluble laminins. (**A**) Blue histograms show expression of alpha subunit of human integrin on HT-29 cells as compared to cells reacted with only 2° antibody (green histograms). Fluorescence intensity is displayed on x-axes and counts are displayed on y-axes. (**B**) HAdV-5 and (**C**) HAdV-41 infection of HT-29 cells in the presence of different concentrations of laminins. The data are represented as % infection of untreated cells. Experiments were performed three times with duplicate samples in each experiment. Error bars in (**B**) and (**C**) represent mean ± SEM. *P of <0.05, **P of <0.01 and ***P of <0.001 versus control. (**D**) Entry of Alexa Fluor labelled 488 in untreated or laminin treated HT-29 cells. Green dots (#) represent internalised virions while yellow/orange dots represent bound/uninternalised virus particles. Ten images of each treatment were taken from two independent experiments. Shown are representative images from each treatment.

In the absence of a human small intestinal cell line, we used HT-29 cells, a human colon cancer cell line that expresses small intestinal markers^26^. HT-29 cells express the laminin-binding integrin subunits α2, α3 and α6 subunit, which can associate with β1 (all) or β4 subunits (α6) and the RGD-binding integrin αV, which associates with β1, 3, 5, 6, and 8. HT-29 cells express no, or very low amounts of α4, α5, α7 and α9 integrin subunits (Fig. 2A), which mirrors the integrin-expression pattern of enterocytes^27^ in the human small intestine. We used recombinant laminin 332 and laminin 511 proteins in our studies, which are ligands for laminin-binding integrins. Laminin 332 contains α3, β3 and γ2 chains and is a ligand for integrins α3β1 and α6β4. Laminin 511 contains α5, β1 and γ1 chains and is a ligand for integrins α2β1, α3β1 and α6β4^28^. Pre-incubation of HT-29 cells with low concentrations (sub μM levels) of laminin 332 reduced HAdV-41 infection efficiently (Fig. 2B). Laminin 511 also reduced HAdV-41 infection but not as efficiently as laminin 332. None of these laminins reduced HAdV-5 infection to the same extent **(**Fig. 2C**)**. However, laminin 332 surprisingly increased HAdV-5 infection by around 60%. Also, pre-incubation of HT-29 cells with vitronectin or fibronectin (RGD-containing integrin ligands) did not affect HAdV-41 infection (Figure S2). These results supported that α2-, α3- and α6-containing integrins are specifically used by HAdV-41 for infection of HT-29 cells. To investigate if one/some of α2, α3 and α6 is/are more essential than other integrins, we infected α6 integrin-deficient HAP-1 cells with HAdV-41. We did not observe reduced infection (data not shown), possibly because HAdV-41 uses multiple integrins.

To establish a direct role of laminin-binding integrins in HAdV-41 internalisation in HT-29 cells, we studied the entry of HAdV-41 on cells that were pre-incubated with laminins. Laminins should bind integrins and make them inaccessible for viral entry. For this, we incubated Alexa Fluor 488-labelled HAdV-41 particles with laminin-treated HT-29 cells for five hours at 37 °C. After incubation, the unfixed cells were stained with an anti-Alexa Flour 488 antibody to identify viral particles on the cell surface as fully internalised particles are protected against the antibody in unfixed cells. The staining procedure was performed on ice to prevent, or minimise, a continued internalisation of bound particles. The data showed a considerable reduction in the number of internalised particles (green particles, labelled with “#” in Fig. 2D) in cells treated with laminins. The ratio of total particles to un-internalised particles was 1.49 to 1 for untreated cells versus 0.88 to 1 and 0.92 to 1 for laminin 332 and laminin 511 treated cells, respectively. This suggests that the integrin binding laminins 332 and 511 prevent entry of HAdV-41 in HT-29 cells.

### Pre-incubation of integrin-expressing cells with soluble, recombinant HAdV-41PB reduces HAdV-41 transduction and infection

**Figure 3:**
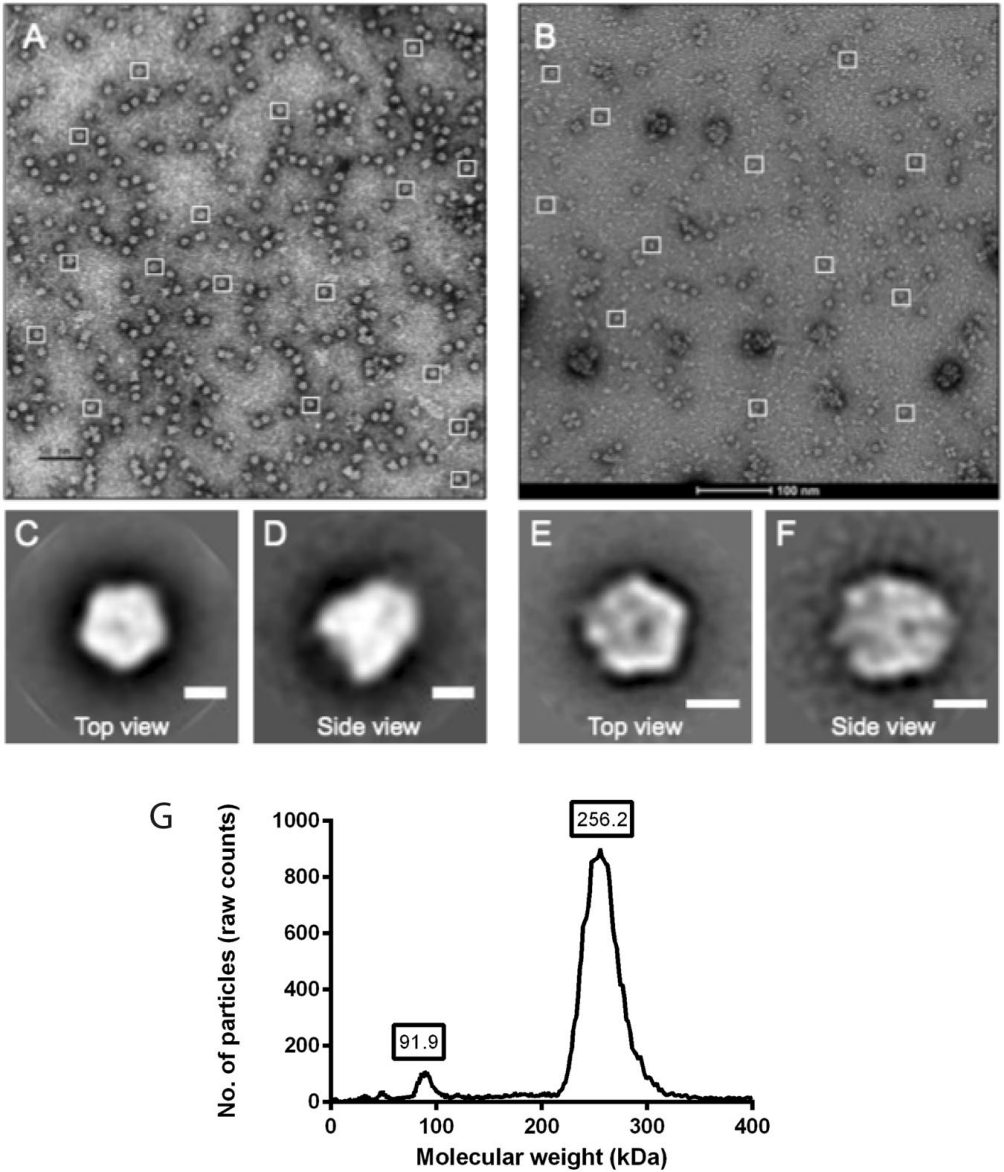
EM and GEMMA analysis of recombinant PBs. Electron micrograph of uranyl acetate negatively stained 41PB (**A**) (pixel size: 2.69 Å) and 5PB (**B**) (pixel size 1.56 Å). The squares mark a number of representative PBs, top and side view, used for single particle analysis using the Scipion software package and the Relion 2D classification algorithm. Bar: 50 nm (**A**) and 100 nm (**B**), 50 Å (**C**–**F**). (**C**) and (**D**) 41PB class averages of top view (579 p.i.) and side view (164 p.i.). (**E**,**F**) 5PB class averages of top view (1201 p.i.) and side view (457 p.i.). (**G**) Gas-phase Electrophoretic Mobility Macromolecule Analysis of 41PB, where the peaks represent the different populations of the PB according to molecular weight.

**Figure 4:**
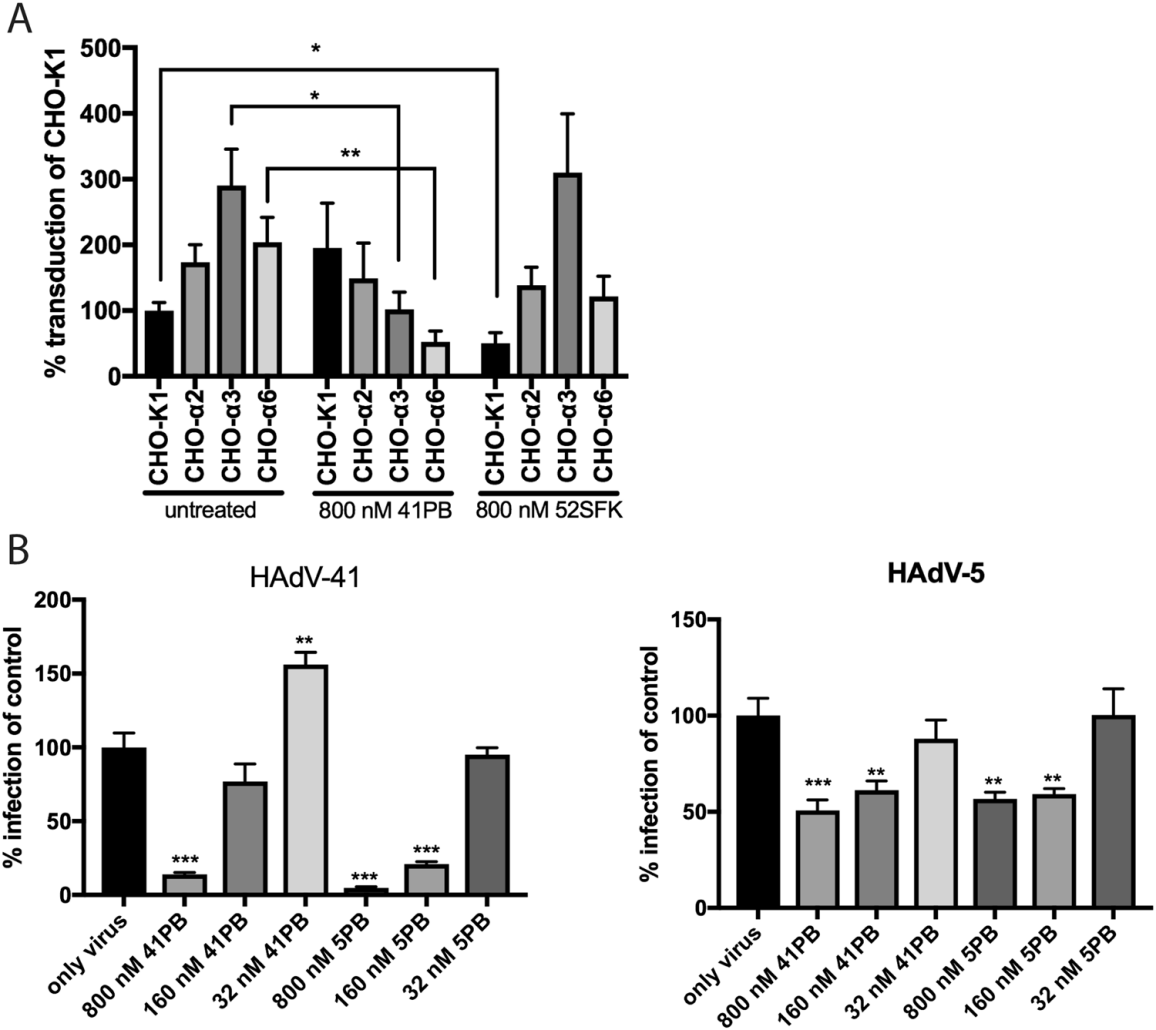
HAdV vector transduction of CHO-cells expressing human alpha integrin subunits and infection of HT-29 cells in presence of soluble 41PB. (**A**) HAdV-41GFP transduction of CHO-cells in the presence of 800 nM 41PB and 800 nM 52SFK. The data are represented as % transduction of control cells (CHO-K1). (**B**) HAdV-41 and HAdV-5 infection of HT-29 cells in the presence of different concentrations of recombinant penton bases of HAdV-41 and HAdV-5 (control protein). The data are represented as % infection of untreated cells. Experiments were performed three times with duplicate samples in each experiment. Error bars represent mean ± SEM. *P of <0.05, **P of <0.01 and ***P of <0.001 versus control.

In order to investigate the function of species F HAdV PBs, we produced these proteins (40PB and 41PB) in a baculovirus system and purified them using liquid chromatography. 5PB was used as a control. The proteins were ~90% pure as determined by SDS-PAGE and by MALDI-TOF MS. We also investigated pentamerisation by electron microscopy (EM) and gas-phase electrophoretic mobility macromolecular analysis (GEMMA) (Fig. 3**)**, the latter to determine molecular weight and multimeric nature of a protein by measuring its diameter in the gas phase^29,30^. These analyses confirmed that the recombinant proteins were predominantly pentameric with the correct structure and molecular weight of ~250 kDa. When CHO-K1, CHO- α2, CHO- α3 and CHO- α6 cells were pre-incubated with soluble 41PB, a reduced transduction of CHO-α3 and CHO-α6 cells was observed (Fig. 4A), but not for the control cell line (CHO-K1) or CHO-α2 cells. This implies that the reduced transduction of CHO-α3 and CHO-α6 cells could be due to the interaction of the PB with these integrins. HAdV-52 short fibre knob protein; 52SFK was used as a control since it is an unrelated protein, which should not have an effect on HAdV-41GFP transduction of CHO-integrin cells. We noted a reduced transduction of control CHO-K1 cells in the presence of 52SFK but we did not observe any reduction in transduction of CHO-α2, CHO-α3 and CHO-α6 cells, suggesting that the inhibitory effect of 41PB on these cells was specific. To study the PB:integrin interaction in a more relevant cell model, HT-29 cells were pre-incubated with 41PB and then infected with HAdV-5 or HAdV-41 virus. HAdV-5 infection was inhibited by 50% at the highest 41PB concentration (800 nM) but HAdV-41 infection was inhibited by more than 85% (Fig. 4B), as compared to controls (no PB). When HT-29 cells were incubated with 5PB, a similar effect on HAdV-41 and HAdV-5 infections was observed. This effect can be explained due to the redundancy in integrin usage by the different types of HAdVs. Reduction in infection of HAdV-41 in the presence of its PB, suggests that the PB protein is needed for efficient infection of HT-29 cells by HAdV-41. This is the first time a function has been established for enteric PBs.

### Role of fibre knob proteins in attachment and entry

**Figure 5:**
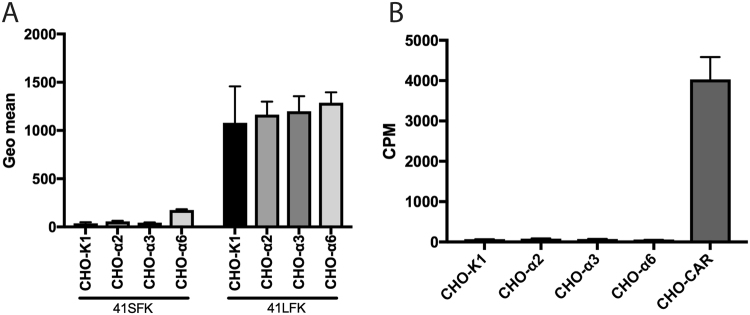
Fibre knob and ^35^S- HAdV-41 virion binding to CHO-cells. (**A**) 41LFK and 41SFK binding to CHO-cells expressing human alpha integrin subunits. Binding is represented by geometric mean. (**B**) ^35^S-labelled HAdV-41 virion binding to CHO-cells expressing human alpha integrin subunits and CHO-CAR cells overexpressing human CAR. Binding is represented by counts per minute (CPM). Experiments were performed twice with duplicate (FK binding) and triplicate (virion binding) samples in each experiment. Error bars represent mean ± SEM.

**Figure 6:**
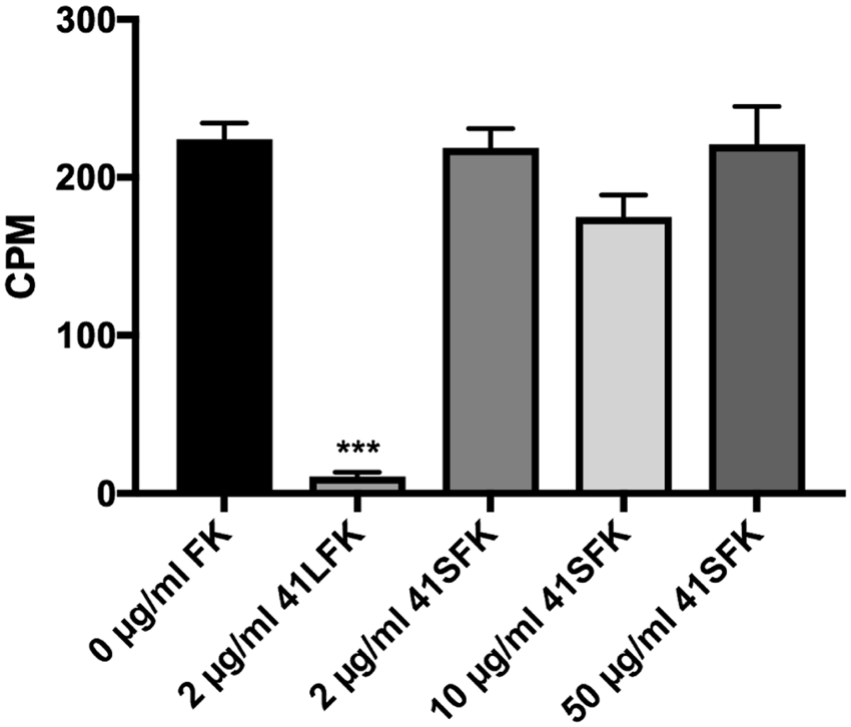
^35^S-labelled HAdV-41 binding to HT-29 cells in presence of soluble FKs. ^35^S-labelled HAdV-41 binding to HT-29 cells preincubated with 41LFK and different concentrations of the 41SFK. Binding is represented by counts per minute (CPM). Experiments were performed three times with duplicate samples in each experiment. Error bars represent mean ± SEM. ***P of < 0.001 versus control.

To determine if the fibre proteins affect transduction of CHO-cells, we performed fibre knob and whole virus binding of HAdV-41 to CHO-K1, CHO-α2, CHO-α3 and CHO-α6 cells. We detected binding of the FKs by flow cytometry using an antibody against the tagged FKs while radiolabelled virus binding was assessed by counts on a microbeta counter. The results show that the binding of the long fibre knob (41LFK) to the control CHO-K1 and to the integrin expressing cells was similar **(**Fig. 5A**)**. The same was observed for the short fibre knob (41SFK). Whole virus only bound to CHO-CAR cells but did not bind to any of the CHO-integrin cells in this experiment (Fig. 5B). Thus, these data show that the increased transduction of the CHO-integrin cells by HAdV-41GFP vector is not dependent on the LF or SF. These data also confirm that transduction is not dependent on the fibre proteins or virus binding to hamster CAR. To establish that the integrins are a co-receptor and do not function as attachment receptors, competition experiments with 41LFK and 41SFK were conducted with ^35^S-labelled HAdV-41 and HT-29 cells. We found that the 41SFK doesn’t block attachment to HT-29 but the 41LFK does **(**Fig. 6**)**, since HT-29 cells express CAR, which is the known attachment receptor.

### HAdV-40PB and -41PB interact with high affinity with α6β4 integrins

**Figure 7:**
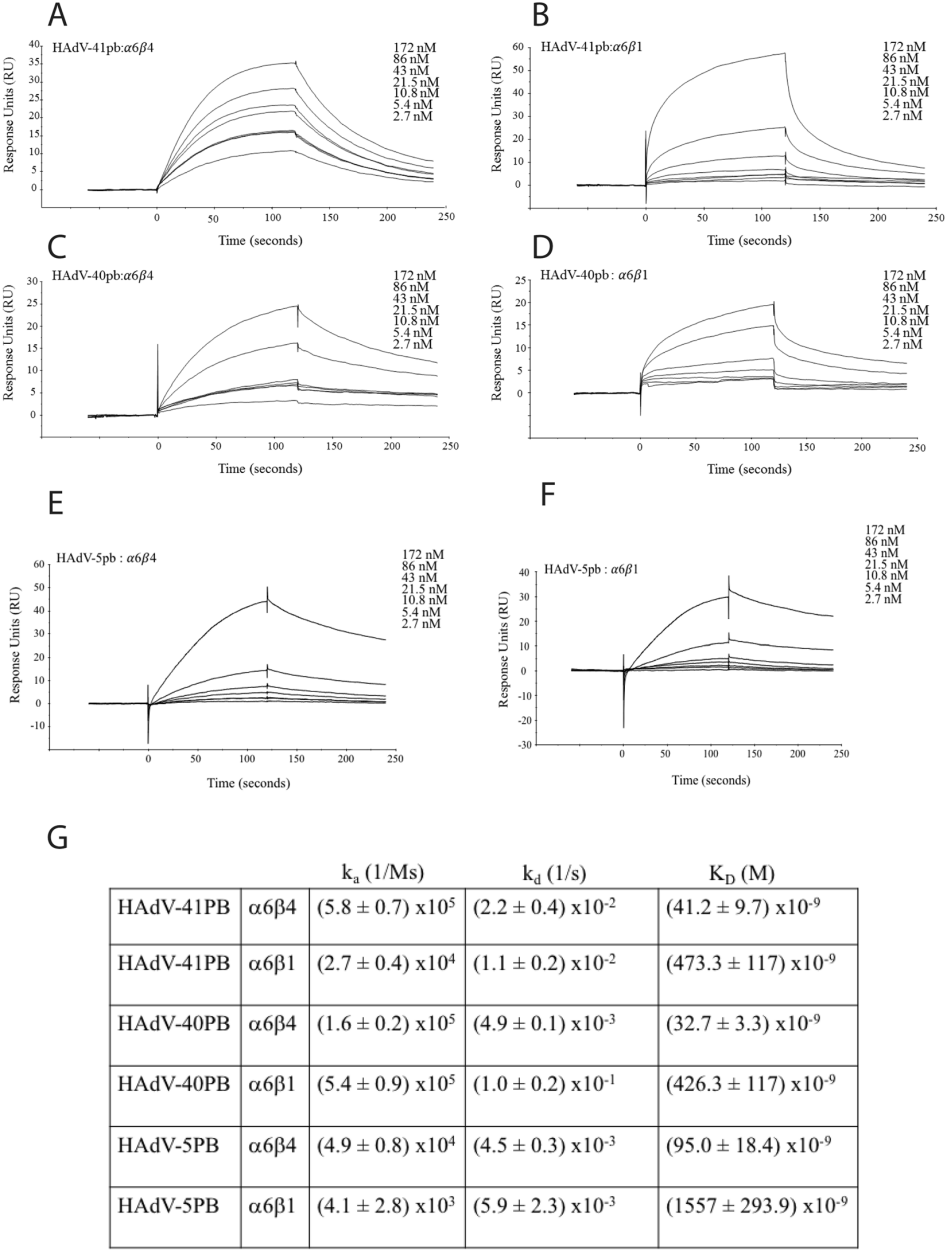
SPR analysis of 41PB, 40PB and 5PB interactions with α6β1 and α6β4 integrins. 41PB (**A**,**B**), 40PB (**C**,**D**) and 5PB (**E**,**F**) were added in two-fold dilution series to immobilised integrins. Results are shown in response units. (**G**) Kinetics of the interaction between the penton bases and integrins. Each interaction was measured four times. Variance is shown as ± SD.

Finally, surface plasmon resonance analysis was performed to confirm direct interaction between immobilised α6-containing integrins (>90% pure as checked by SDS-PAGE) and the recombinant PB proteins (added in solution) and to determine affinity and kinetics of these interactions. Both 40PB and 41PB bound with high affinity (30–40 nM range) to α6β4 and with approximately ten-fold lower affinity to α6β1 (Fig. 7). With a few exceptions, the kinetics of the interactions were relatively similar with most association rates in the 10^5^ M^−1^ s^−1^ range and most dissociation rates in the 10^−2^ s^−1^ (or close to) range. However, the lower affinities for α6β1 resulted from either a markedly slower association of 41PB (low 10^4^ M^−1^ s^−1^ range) or from a markedly faster dissociation of 40PB (low 10^−1^ s^−1^ range). The affinity of these interactions is in the range determined by previous studies for 2PB:integrin interactions^14,31^. 5PB affinity to α6β4 and α6β1 was approximately three times lower. This provided further evidence that there is a direct interaction between the enteric HAdV PB proteins and α6- containing integrins.

## Discussion

Here we found that enteric species F HAdVs may use the laminin-binding α2-, α3-, and α6-containing integrins. Studies from other groups have shown that several, RGD-binding integrins such as αVβ1, αVβ3, αVβ5, and α5β1, as well as the laminin-binding integrin α3β1^14,15,18,19,32,33^ are engaged in entry and/or endosomal release of other HAdVs. Our data suggest that the enteric species F HAdVs are more promiscuous and use a fundamentally different repertoire of integrins than other HAdVs, which appears surprising, since the tropism of this virus is strictly limited to the intestinal tract. As per our knowledge, there have been no studies to date explaining the function of the PB of the enteric species F HAdVs, which lack the otherwise conserved RGD motif. We hypothesised that these viruses interact with integrins other than the RGD-binding integrins and through RGD-independent mechanisms. Whereas the RGD motif is the most well documented integrin-binding motif, there are many other motifs known to interact with other integrins. For example, it has also been shown that SIKVAV^34^ and a glutamic acid residue in the third position from the C-terminus of laminin γ chains (I**E**K and L**E**Q) are involved in integrin binding^35^. 40PB and 41PB (but not the PBs of other HAdVs) contain amino acid motifs resembling those of integrin-binding laminins: ASIK/IEQ (HAdV-40) and ASIQK/IEK (HAdV-41)^22,35–37^, both motifs being positioned in the loop corresponding to the RGD loop. To identify whether enteric HAdVs employ specific integrins as co-receptors, we used a library of CHO cells that overexpress various human integrins. CHO cells that express human integrin alpha have been used in many studies^25,38,39^ which defined the binding specificity of integrins to many new ligands including several viruses. To date, human alpha/hamster beta integrin dimers have been shown to be identical to their human counterpart in ligand specificity. Since these cells are refractory to wild type HAdV infection, we used a HAdV-41GFP vector and quantified transduction. Cells expressing human α2, α3, α5, α6, α8 and α9 subunits were transduced to a higher degree than their corresponding control CHO cells. The use of multitude of integrins revealed in this experiment suggested a more promiscuous usage of integrins by HAdV-41 (and probably also HAdV-40) as compared by other HAdVs. The identity of the target cells where enteric HAdVs replicate has not yet been established, but non-human enteric AdVs such as avian, murine and porcine AdVs replicate in the small intestinal epithelial cells of their hosts^40–42^. In humans, these cells express various integrins, which have different distributions throughout the intestine **(**Table 1**)**. RGD-binding integrins such as αV have not been detected in the intestinal epithelium of the small intestine^43^. The presence and distribution of laminin-binding integrins in the human intestine and the presence of motifs in the PB of enteric HAdVs that potentially interact with laminin-binding integrins lead us to investigate the impact of these integrins in further detail. When HT-29 cells were pre-incubated with soluble laminin-332 and -511, a dose-dependent decrease in infection of WT HAdV-41 was observed. The effect was less pronounced and dosage independent for HAdV-5, which was used as a reference. The increased infection of HAdV-5 with the highest concentration of laminin 332 is an interesting observation, which we cannot fully explain. We hypothesise that under certain conditions, laminins can function as a bridge and generate indirect binding of virions to cells, as has been suggested in the case of fibronectin and HAdV-37^33^. Laminins also bind to cellular molecules other than integrins, such as α-dystroglycan, 67 kDa laminin-binding protein, galactosyltransferase, and syndecan-1^28^, which we did not evaluate further. We cannot exclude that the effect of laminins that we observed is due to interactions with these molecules, but taken together with the other data presented here, these results support that HAdV-41 makes use of one or more of the laminin-binding integrins for infection, rather than any of these non-integrins. We also identified the participation of laminin-binding integrins in the entry of HT-29 cells by HAdV-41, where HT-29 cells treated with soluble laminin clearly had fewer internalised viral particles as compared to untreated cells. These date indicate that the laminin-binding integrins play a role in virus entry or internalisation. Next, soluble recombinant 40PB and 41PB were produced in the baculovirus system in which PBs of other types of HAdVs^22,44^ have been purified previously for structural and functional studies. 41PB reduced HAdV-41 transduction of CHO-α3 and CHO-α6 cells, but not the transduction of CHO control cells or CHO-α2 cells. Since α3 and α6 are expressed on the villus and α2 is mainly expressed in crypts these results suggest that HAdV-41 may have a preference to infect cells on the villus rather than the crypt. Upon co-incubation with 52SFK we observed a small but statistically significant (P value = 0.0396) decreased transduction of the control cells (CHO-K1). However, the control protein did not affect the transduction of the integrin-expressing cells. A significant (P value for HAdV-41 = <0.0001 and for HAdV-5 = 0.0009) reduction in infection was seen for both viruses when HT-29 cells were pre-treated with soluble PB and then infected with wild type HAdV-41 or HAdV-5. We speculate that the reduced infection of HAdV-5 in the presence of 41PB and vice-versa is because integrins used by HAdV-5 overlaps with some of those used by HAdV-41, such as α3β1^32^. We also observed that HAdV-41GFP transduced CHO cells that overexpress RGD binding integrins such as α5- and α8-containing integrins, and hence it could be that 41PB reduced infection of HAdV-5 through interactions with these integrins. Since HT-29 cells express all the candidate laminin-binding integrins such as α2, α3 and α6, it is difficult to conclude from these experiments if one or some of these integrins is more essential than others. To further investigate the importance of individual integrins we infected α6 integrin knockout HAP1 cells. We did not observe a reduction in infection by HAdV-41 as compared to the control cells. HAP1 and HT-29 cells express many different integrin types including α2, α3 and β4. This feature together with the fact that integrin usage by HAdVs is redundant, it would be an impractical task to interfere with multiple integrins by knockouts, soluble integrins or anti-integrin antibodies. We performed HAdV-41 infection of HT-29 cells and CHO-integrin cells in the presence of soluble integrins and anti-integrin antibodies but only observed minor effects (data not shown).

**Table 1:** Examples of LDV-, RGD-, collagen- and laminin-binding integrins, their ligands and their presence/location in the small intestine.

Integrin (integrin group)	Ligand^a^	Presence and location in the small intestine^b^
α1β1 (collagen/laminin-binding)	Collagen	Unknown
α2β1 (collagen/laminin-binding)	Collagen, laminin 511	Predominantly in the crypts
α3β1 (laminin-binding)	Laminin 332, 511 and 521	Mainly on the villus
α4β1 and α4β7 (LDV-binding)	VCAM-1, MAdCAM-1	Deeper layers of the epithelial tissue involved in epithelial:lymphocyte interactions
α5β1 (RGD-binding)	Fibronectin, vitronectin	Base of crypt and villus cells
α6β1 and α6β4 (laminin-binding)	Laminin 111, 332, 511 and 521	Uniformly distributed from the bottom of the crypts to tip of the villus
α7β1 (laminin-binding)	Laminin 111, 211 and 221	Upper region of crypts and lower region of villus
α8β1 (RGD-binding)	Fibronectin, vitronectin, tenascin	Expressed in the crypts
α9β1 (LDV-binding)	Fibronectin, tenascin-C, osteopontin	Transiently expressed by cells in the crypts
α10β1 (collagen-binding)	Collagen	Unknown
α11β1 (collagen-binding)	Collagen	Unknown
αvβ1, αvβ3, αvβ5 (RGD-binding)	Fibronectin, vitronectin, osteopontin	Undetected

^a^List of selected, known ligands, ^b^‘unknown’ denotes unknown presence or location.

41LFK bound equally to each of the CHO cells (parental and integrin-expressing). We observed the same with the 41SFK. Whole virus only bound to CHO cells expressing human CAR and not to integrin expressing CHO cells, which shows that integrins are not required for binding. On HT-29 cells, only the 41LFK blocked attachment of HAdV-41 and not the 41SFK, which confirms CAR, and not integrins, as the attachment molecule. These data, taken together with the results from CHO cell transduction experiments, suggest that the PB protein (and not the fibre proteins) is responsible for increased transduction of the CHO-integrin cells. This signifies that laminin-binding integrins function as secondary or accessory receptor and not as primary attachment receptors for HAdV-41. Furthermore, SPR studies with α6β1 or α6β4 and 5PB or 41PB showed that the affinity of 41PB was three times higher than that of 5PB and that the affinity of this interaction is similar to the affinities of RGD-equipped penton base:integrin interactions seen in previous studies^14,31^.

In conclusion, since the small intestinal epithelium does not express, to the best of our knowledge, RGD-recognising αV integrins^27^, it is likely that the enteric species F HAdVs have adapted to use other integrins that are present on intestinal epithelial cells. Laminin-binding integrins, especially α6β4, are predominantly expressed on the basal surface of intestinal epithelial enterocytes^45^. When these cells migrate from the crypt and are shed from the villus, gaps are created between the enterocytes^46^, which might expose integrins as well as lateral and basal CAR, the attachment molecule for the LFs of species F HAdVs. Analogous to the infection route of other HAdVs, we envision that the LF binds CAR, tethering the virion to the cell and allowing subsequent interactions between PB and integrins to occur, which results in entry and infection. The function of the RGD-lacking PB proteins of enteric species F HAdVs has been an enigma. It has been suggested that the entry mechanism for enteric species F HAdVs is different from that of other HAdVs *i.e*., the SF replaces the PB as mediators of cellular entry or entry is integrin-independent^5,24^. Based on the results presented here we propose that 41PB are used during virus entry. However, we cannot exclude the possibility that the SF also contributes to entry. Specifically, we propose that HAdV-41 and probably also HAdV-40 use laminin-binding integrins as well as other integrins as co-receptors, but do not use the αV-containing integrins that are used by multiple other HAdVs.

## Materials and Methods

### Cells, viruses, vectors and antibodies

*Cells:* Chinese Hamster Ovary (CHO)-K1 cells were grown in Ham’s F12 Nutrient mix (Gibco); CHO-B2 cells were grown in Dulbecco’s modified Eagle’s medium (Sigma-Aldrich); and CHO-α7 cells (from GenScript) were grown in Ham’s F12K nutrient mix (Kaighn’s modification, Gibco) with 4 µg/ml of puromycin (Gibco). The above cell media were supplemented with 10% fetal bovine serum (FBS, Thermo Fisher Scientific), 20 mM HEPES buffer (Sigma-Aldrich) and 20 U/ml penicillin + 20 µg/ml streptomycin (PEST, Thermo Fisher Scientific). CHO-K1 was used for generation of CHO-α2^47^, CHO-α3^39^, CHO-α6^48^, CHO-α7, CHO-α8^49^ and CHO-α9^50^ whereas CHO-B2 was used for generation of CHO-αV^51^, CHO-α4β1^52^, CHO-α4β7^53^ and CHO-α5^51^. The different CHO cells were grown as described^39,47–53^. HT-29 cells (colorectal adenocarcinoma cells) (gift from Dr. Marie-Louise Hammarström) were grown in McCoy’s 5 A medium (Gibco) with PEST and 10% FBS. All the above cells were cultured at 37 °C in the corresponding media. *Spodoptera frugiperda* Sf9 insect cells (Gibco) were grown and maintained at 28 °C either as adherent or suspension cultures in Sf900 II SFM serum-free media (Gibco) supplemented with PEST. *Virus:* Species F HAdV-41 (strain Tak) (kindly provided by Dr. Alistair H. Kidd) and species C HAdV-5 (strain adenoid 75; ATCC) were produced in A549 cells with or without ^35^S-labeling as described^54^ but the virions were eluted in sterile phosphate buffered saline (PBS) instead of sterile DMEM, after desalting CsCl, using NAP columns (GE Healthcare). The virions were stored at −80 °C in PBS with 10% glycerol. HAdV-41 was labelled using the Alexa Fluor 488 Microscale Protein Labeling Kit (Thermo Fischer Scientific) according to the manufacturer’s protocol. After the labelling reaction, the conjugate was purified by dialysis (Slide-A-Lyser dialysis cassette with 10000 MWCO, Thermo Fischer Scientific) against one litre of PBS overnight followed by 2–3 hours of dialysis in one litre PBS with 10% glycerol the next day. *Vectors:* HAdV-41 GFP vector was produced as described^55^ and HAdV-5 GFP vector was purchased from Vector Development Lab. *Antibodies* used in flow cytometry and western blot were P2W7 (anti-αV, Abcam), P1E6 (anti-α2, Merck Millipore), ASC-1 (anti-α3, Merck Millipore), 44H6 (anti-α4, Abcam), P1D6 (anti-α5, Abcam), GoH3 (anti-α6, Abcam), 3C12 (anti-α7, LifeSpan BioSciences), clone # 481709 (anti-α8, R&D systems), Y9A2 (anti-α9, Acris Antibodies), 422325 (anti-β1, R&D systems), P5D2 (anti-β4, R&D systems), anti-actin antibody (Sigma-Aldrich), RmcB (anti-CAR, Merck), anti-RGS His (recognises N-terminal His tags with the epitope RGSHHHH; Qiagen), anti-mouse IgG Alexa Fluor 488 (Life technologies) and anti-rat IgG FITC antibody (Dako Cytomation). Serotype-specific rabbit polyclonal antisera against HAdV-41 and HAdV-5 used in infection experiments were a kind gift from Dr. Göran Wadell^56^.

### Recombinant proteins

*Integrin ligands* used in infection experiments were laminin 332 and laminin 511 (Biolamina), vitronectin (R&D) and fibronectin (Roche). *Recombinant human integrins* used for surface plasmon resonance (SPR) experiments (α6β4 and α6β1) were purchased from R&D Systems. *40PB, 41PB and 5PB:* Full length 40PB, 41PB or 5PB DNA was cloned in pFastBac HT A vector (providing hexa-histidine tag to N-terminus of expressed protein) and transformed into *E.coli* DH10Bac. The recombinant bacmids were further analysed by PCR according to the Bac-to-Bac Baculovirus Expression System kit from Invitrogen. Sf9 (*Spodoptera frugiperda*) cells were transfected with the bacmid DNA and incubated at 28 °C for 96 hours, generating a passage 1 (P1) baculovirus stock that was then used to generate high-titre P2 stock. 4.3 × 10^5^ cells/ml of Sf9 cells were infected at a MOI of 5 with the P2 viral stock and the cells were incubated at 28 °C for four days under shaking conditions. After incubation, the cells were lysed and purified first with Ni-NTA agarose column (Qiagen) and then with a HiTrap Q-sepharose column (GE Healthcare) using an ÄKTA liquid chromatography system (GE Healthcare). The soluble recombinant proteins were then stored in PBS with 10% glycerol in −20 °C. *41SFK and 41LFK:* Cloning and purification of 41SFK (amino acids 215 to 387) and 41LFK (amino acids 363 to 562) was done as described in^13^.

### Integrin/CAR expression and fibre knob binding assay

Flow cytometry analysis was performed to check integrin expression on CHO-integrin cells and HT-29 cells and to check expression of human CAR on CHO-integrin cells. CHO-B2 and -K1 control cells, corresponding integrin-expressing cells (except CHO-α8), CHO-CAR and HT-29 cells were detached with PBS-EDTA, counted and then allowed to recover in growth media at 37 °C. After one hour, the cells were added to a V-bottom 96-well plate (2 × 10^5^ cells/well) and washed once with FACS buffer (PBS with 2% FBS). Integrin-recognising or anti-CAR monoclonal antibodies were diluted 1:500 or 1:40 respectively in FACS buffer and incubated with the cells for 30 minutes at 4 °C. Unbound antibodies were washed away with FACS buffer and the cells were then incubated with Alexa Fluor 488 donkey anti-mouse IgG (diluted 1:1000 in FACS buffer) or rabbit anti-rat IgG FITC antibody (diluted 1:40 in FACS buffer) for 30 minutes at 4 °C. After incubation with the secondary antibody, the cells were washed with FACS buffer and analysed on the FACSLSRII cytometer (Becton Dickinson). The results were analysed with FACSDiva software (Becton Dickinson). To determine FK binding on the CHO-integrin cells (CHO-K1, CHO-α2, CHO-α3 and CHO-α6 cells), 10 μg/ml of 41LFK and 41SFK diluted in 100 μl serum-free media were added to the cells for one hour on ice, washed and then stained with an anti- RGS His mouse monoclonal antibody (diluted 1:200 in FACS buffer) for 30 minutes. After washing unbound primary antibody, the same method was followed as with the integrin/CAR expression samples.

### Western blot

CHO-K1 or CHO-α8 cells were detached with PBS-EDTA. The cells were washed once with PBS-EDTA and then suspended in lysis buffer (10 mM Tris-HCl, 150 mM NaCl, 0.5 mM EDTA, 1% Triton X-100, pH 7.5). Cell debris were pelleted by centrifugation at 12000 RPM for 10 minutes at 4 °C. Equal amounts (determined by Pierce BCA protein Assay Kit, Thermo Fisher Scientific) of each cell supernatant was resolved on a 12% Bis-Tris denaturing gel (NuPAGE, Invitrogen) and transferred to a nitrocellulose membrane (Bio-Rad Laboratories). The membrane was blocked with 5% non-fat dry milk in PBS-T (PBS supplemented with 0.05% Tween 20) and incubated with an anti-human α8 integrin monoclonal antibody (diluted 1:1500) or with anti-actin antibody (diluted 1:3000). The antibodies were diluted in 5% non-fat dry milk in PBS-T. After three ten-minute washes with PBS-T, the membrane was incubated with a 1:1000 diluted HRP conjugated secondary antibody (Dako Cytomation) in PBS-T with 2.5% non-fat dry milk. Expression of human α8-integrin was detected by chemiluminescence using SuperSignal West Femto (Thermo Fisher Scientific) and visualised using the multipurpose CCD camera system FujiFilm LAS-4000.

### HAdV vector transduction

CHO cells were grown as monolayers in 96-well clear bottom plates (Greiner bio-one). The cells were washed three times with serum-free medium before pre-incubation with/without recombinant, 41PB, or with recombinant, soluble 52SFK at 4 °C for one hour. HAdV-5GFP vector (11250 vp/cell) or HAdV-41GFP vector (100000 vp/cell) was then incubated with the cells for two hours on ice. After incubation, the cells were washed three times with serum-free medium and then incubated at 37 °C for 72 hours in maintenance medium (growth medium with 1% FBS). Subsequently, the cells were washed once with PBS and then examined in a fluorescence plate reader (TROPHOS, Luminy Biotech Enterprises) in PBS. Numbers of infected cells were calculated using the Tina program of the TROPHOS. The results are expressed as % transduction of control cells (either CHO-K1 or CHO-B2), where GFP signal per well is counted.

### Electron Microscopy

Negative staining EM: Samples were applied to carbon-coated copper-grids and stained with 1.5% Uranyl Acetate (UA). The 41PB sample was analysed with a JEOL-1230 TEM-Microscope (JEOL, Japan) at 80 kV and micrographs were acquired with a Orius 830 2k × 2k CCD camera using the digital micrograph software (Gatan, US) and a pixel size of 2.69 Å on the objective scale. The 5PB sample was analysed with a Talos 120 C TEM-Microscope (FEI, The Netherlands) operating at 120 kV and micrographs were acquired with a Ceta 16 M CCD camera (FEI) using the TEM Image & Analysis software ver. 4.15 (FEI) and a pixel size of 1.56 Å on the objective scale.

### EM Image Processing and Modeling

Particle picking and data processing were performed with the Scipion software package^57^. Datasets with more than 1600 extracted particle images (p.i.) of PBs (41PB: 1637 p.i and 5PB: 2419 p.i.) were processed and 2D classified into 2D classes using the Relion (2D classification) algorithm^58–60^. 41PB p.i. were classified into 6 classes, a good symmetrical class of a top view and a side view were selected (Top: 579 p.i.; Side: 164 p.i.). 5PB p.i. were classified into 70 classes, after exclusion of p.i. in non-representative classes, further 2D classification into 10 classes (1836 p.i.) was performed. Additional exclusion of p.i. in non-representative classes generated 2 good symmetrical 2D classes of a top view and a side view of 5PB (Top: 1201 p.i.; Side: 457 p.i.).

### Gas phase Electrophoretic Mobility Macromolecular Analysis (GEMMA)

Buffer was changed from PBS (41PB was stored in PBS) to 20 mM ammonium acetate buffer, pH 7.8 containing 0.005% (v/v) Tween 20 using G-25 columns (GE Healthcare) since the sample should not contain non-volatile salts. The PB protein in 20 mM ammonium acetate was scanned five times (120 seconds per scan) with a capillary pressure of 3.7 bar and a diameter range of 2.55–255 nm. For molecular mass calculations, a particle density of 0.58 g/cm^3^ was used^61^.

### Infection assay

HT-29 cells were grown as monolayers in 96-well clear bottom plates. The cells were washed three times with serum-free medium and treated with or without different concentrations of (a) recombinant laminin 332 or laminin 511 or (b) recombinant 41PB or 5PB (negative control) for one hour on ice. HAdV-5 and HAdV-41 were incubated with the cells for one hour on ice after a wash. Following incubation, the cells were washed three times with serum-free medium and then incubated at 37 °C for 44 hours in maintenance medium (growth medium with 1% FBS). Next, the cells were washed once with PBS and then fixed with ice-cold methanol at −20 °C for 10 minutes. The cells were then stained with polyclonal rabbit anti-HAdV-5 (1:10 000) or anti-HAdV-41 (1:300) diluted in PBS for 30 minutes at room temperature. After two 15-minute washes with PBS, the cells were incubated with goat anti-rabbit IgG (H + L) secondary antibody with an Alexa Fluor 647 conjugate (1:250) diluted in PBS for 30 minutes at room temperature. Pictures were taken and numbers of infected cells were calculated using the Tina program of the TROPHOS after two 15-minute washes with PBS.

### Internalisation assay

HT-29 cells were grown as monolayers in each well of an 8-well tissue culture chamber on a slide. The cells were washed with PBS and treated with or without 0.1 µM laminin 332 or laminin 511 for one hour on ice. Alexa Flour 488 labelled HAdV-41 was diluted 1:100 in McCoy’s 5A media with 3% FBS and added to the cells without washing away the laminins. The cells were incubated with the virus for five hours at 37 °C to facilitate entry. After incubation, the cells were rinsed once with ice cold PBS +10% FBS and kept on ice for 15 minutes prior to staining. All subsequent steps where performed on ice, unless otherwise stated. First, un-internalised virus particles were stained using a rabbit polyclonal antibody raised against the Alexa Fluor 488 dye (Thermo Fisher Scientific), diluted 1:250 in PBS +10% FBS. After one hour of slow rocking, the cells were washed three times with cold PBS +10% FBS and a goat anti rabbit secondary Alexa Fluor 568 conjugated antibody (Thermo Fisher Scientific) was added diluted 1:1000 in PBS +10% FBS and allowed to bind for one hour. Finally, the cells were washed three times with PBS +10% FBS and fixed with 4% PFA at room temperature for 20 minutes, rinsed twice with PBS, and the nucleus stained by Hoescht 33342. The slides were mounted with ProLong Gold Antifade and imaged using a Nikon confocal microscope. A total of 30 images were taken using a 100x lens and quantification was performed using the analyse particle routine in ImageJ^62^. The images were used to calculate a ratio between the total number of viral particles (green/Alexa Fluor 488) and uninternalised particles (red/Alexa Fluor 568 and green/Alexa Fluor 488).

### ^35^S-labelled virion binding assay

CHO-K1, CHO-α2, CHO-α3, CHO-α6 and CHO-CAR cells were detached with PBS-EDTA, reactivated in growth media for one hour at 37 °C (in solution), pelleted in 96 well plates (2 × 10^5^ cells/well) and washed with binding buffer (BB: Dulbecco’s modified Eagle medium supplemented with HEPES and PEST). ^35^S-labelled virions (2 × 10^9^ virions diluted in BB, 100 μl/sample) were added to the cells and incubated for one hour on ice. Unbound virions were washed away with BB and the cell-associated radioactivity was measured in a Wallac 1450 Microbeta liquid scintillation counter (Perkin-Elmer). To study effect of soluble FKs on binding of ^35^S-labelled HAdV-41, cells were incubated with different concentrations of 41SFK and 2 µg/ml of 41LFK after reactivating cells. The unbound fibre knobs were washed away with BB before addition of virions.

### Surface Plasmon Resonance

SPR experiments were performed using Biacore T200 optical biosensors (GE Healthcare). Standard EDC/NHS coupling was used to covalently immobilise the integrin α6β4 and α6β1 to CM5 sensor chips for 420 seconds at a flow rate of 10 µl/minute using a 40 µg/ml integrin concentration in 10 mM sodium acetate, pH 4.0. Immobilisation density was approximately 16000 response units (RU). For each Biacore kinetic experiment, a series of seven PB concentrations serially diluted 2-fold was prepared in the running buffer (10 mM HEPES, 150 mM NaCl, 1 mM CaCl_2_, 1 mM MgCl_2_ with 0.05% (v/v) surfactant P20, pH 7.4) and injected for 120 seconds at 30 µl/minute followed by 120 seconds of dissociation. All covalent surfaces were regenerated with one 30 second pulse of 10 mM glycine–HCl (pH 1.5, GE Healthcare). All Biacore kinetic experiments were conducted at 25 °C. Biacore sensorgrams and binding affinities were calculated and processed using Biacore T200 evaluation software (version 2.0, GE Healthcare).

### Statistical analysis

All experiments were performed two or three times with duplicate or triplicate samples in each experiment. The results are shown as ± standard error of mean (SEM) and t-test was performed using GraphPad Prism 7 for Mac OS X. P-values < 0.05 were considered statistically significant. For SPR experiments, variance is denoted as ± standard deviation (SD).

## Electronic supplementary material

Dataset 1
