# Supplementary material for: Enteric Species F Human Adenoviruses use Laminin-Binding Integrins as Co-Receptors for Infection of Ht-29 Cells
Source: Sci Rep. 2018 Jul 3;8:10019. doi: 10.1038/s41598-018-28255-7 (PMC6030200; doi:10.1038/s41598-018-28255-7)
Supplement: Supplementary file 1 — Dataset 1 [file 41598_2018_28255_MOESM1_ESM.pdf]

ENTERIC SPECIES F HUMAN ADENOVIRUSES USE LAMININ-BINDING INTEGRINS AS CO-RECEPTORS FOR INFECTION OF HT-29 CELLS

Anandi Rajan[a], B. David Persson[a+], Lars Frängsmyr[a+], Annelie Olofsson[b], Linda Sandblad[b], Jyrki Heino[c], Yoshikazu Takada[d], A. Paul Mould[e], Lynn M Schnapp[f], Jason Gall[g], Niklas Arnberg[a*]

[a]Department of Clinical Microbiology/ Division of Virology and the Laboratory for Molecular Infection Medicine Sweden, Umeå University, Sweden;

[b]Department of Molecular Biology, Umeå University, Sweden;

[c]Department of Biochemistry, University of Turku, Finland;

[d]Department of Dermatology, Biochemistry and Molecular Medicine, UC Davis School of Medicine, California, USA;

[e]Biomolecular Analysis Core Facility, Faculty of Biology, Medicine and Health, University of Manchester, United Kingdom;

[f]Division of Pulmonary, Critical Care, Allergy and Sleep Medicine, Medical University of South Carolina, Charleston, USA;

[g]Vaccine Research Center (VRC), NIAID, NIH, Bethesda, USA

Running Head: Enteric HAdVs and laminin-binding integrins

*Address correspondence to Niklas Arnberg, niklas.arnberg@umu.se

+ These authors contributed equally to this work.

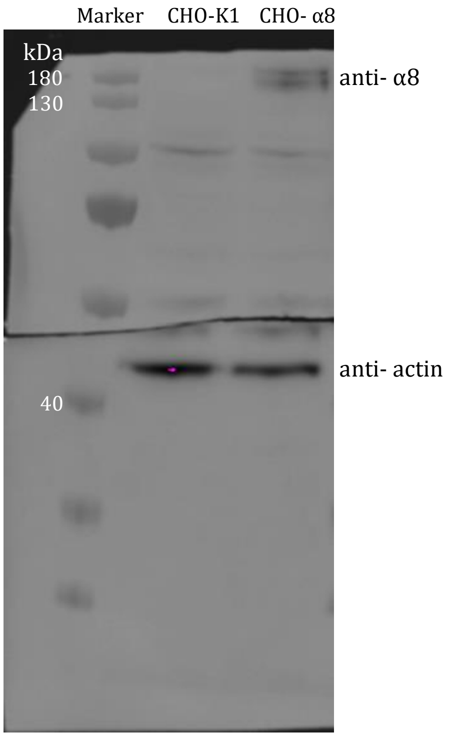

Figure S1: **Expression of human α8 integrin.** Full length blot of analysis of α8 integrin in transfected CHO cells and control CHO-K1 cells.

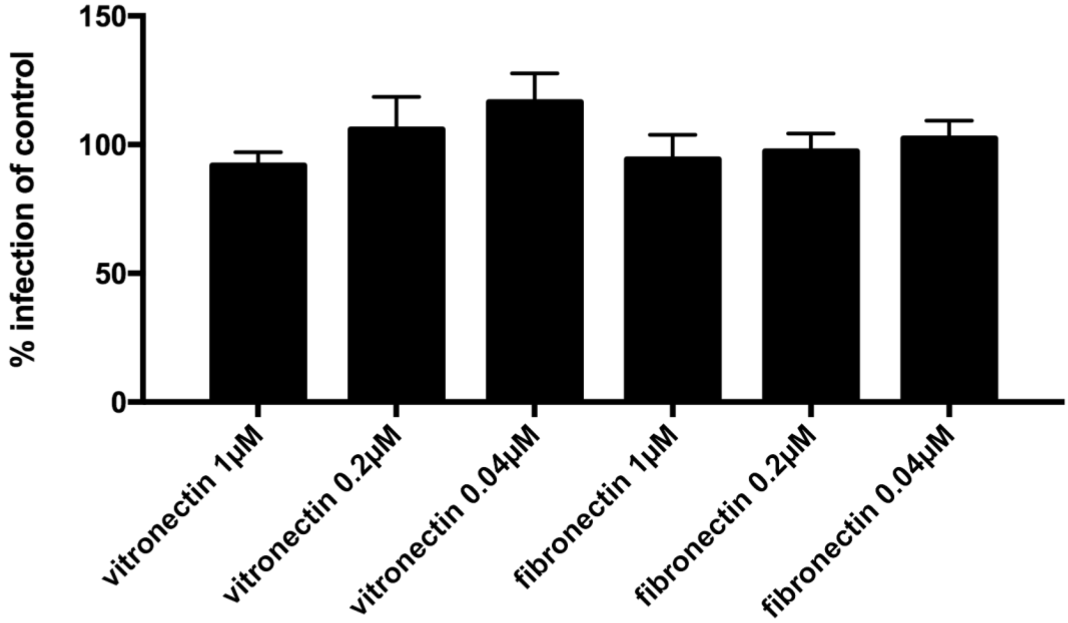

Figure S2: **HAdV-41 infection of HT-29 cells in presence of soluble vitronectin and fibronectin.** HAdV-41 infection of HT-29 cells in the presence of different concentrations of vitronectin and fibronectin. The data is represented as % infection of untreated cells. Experiments were performed twice with duplicate samples in each experiment. Error bars represent mean ± SEM.
